# A combination of unenhanced CT-derived features predicts renal parenchymal involvement of immunoglobulin G4-related disease

**DOI:** 10.1186/s41747-026-00768-1

**Published:** 2026-06-25

**Authors:** Jun Inui, Hidenori Amaike, Masatoshi Kanda, Naoya Yama, Hiroki Takahashi, Masamitsu Hatakenaka

**Affiliations:** 1 Division of Diagnostic Radiology, Department of Radiology, School of Medicine, Sapporo Medical University, Sapporo, Japan; 2 Division of Rheumatology and Clinical Immunology, Department of Internal Medicine, School of Medicine, Sapporo Medical University, Sapporo, Japan

**Keywords:** ImmunoglobulinG4-related disease, Kidney, Radiomics, Renal insufficiency, Tomography (x-ray computed)

## Abstract

**Objective:**

To predict renal parenchymal involvement in patients with immunoglobulin G4-related disease (IgG4-RD) using texture- and volume-based analysis of unenhanced computed tomography (CT) images.

**Materials and methods:**

Fifty-nine patients with IgG4-RD, diagnosed according to American College of Rheumatology/ European Alliance of Associations for Rheumatology classification criteria, who underwent both unenhanced CT and contrast-enhanced (CE)-CT) covering the kidneys before treatment (May 2007–July 2023), were enrolled. After excluding 15 cases, 44 patients remained for analysis. Clinical variables (sex, age, body weight, estimated glomerular filtration rate) and kidney volume-related and texture metrics extracted from unenhanced CT were analyzed for inter-reader repeatability and correlation with renal parenchymal involvement.

**Results:**

Kidney involvement of IgG4-RD was determined on CE-CT by a rheumatologist and a radiologist in consensus, resulting in 23 cases with and 21 cases without renal parenchymal involvement. One radiologist and a radiology resident independently extracted metrics from unenhanced CT. Two metrics, kidney volume per body weight and gray-level co-occurrence matrix (GLCM) cluster shade, showed high repeatability (intraclass correlation coefficients 0.997 and 0.762, respectively) and significant association with renal parenchymal involvement (area under the receiver operating characteristic curve [AUROC] > 0.75). Binary logistic regression combining these two metrics predicted renal parenchymal involvement with AUROCs of 0.872 and 0.896 for the two readers, respectively.

**Conclusion:**

A combination of kidney volume per body weight and GLCM cluster shade derived from unenhanced CT can predict renal parenchymal involvement in IgG4-RD.

**Relevance statement:**

The combination of two unenhanced CT-related metrics−kidney volume per body weight and GLCM cluster shade−can predict renal parenchymal involvement of immunoglobulin G4-related disease, with the AUROCs for the two readers being 0.872 and 0.896, respectively.

**Key Points:**

- Unenhanced CT has been considered not to detect renal parenchymal involvement of IgG4-RD.
- By using image segmentation and texture analysis techniques, renal parenchymal involvement could be predicted in IgG4-RD.
- A combination of kidney volume per body weight and GLCM cluster shade could predict renal parenchymal involvement with high AUROC.

**Graphical Abstract:**

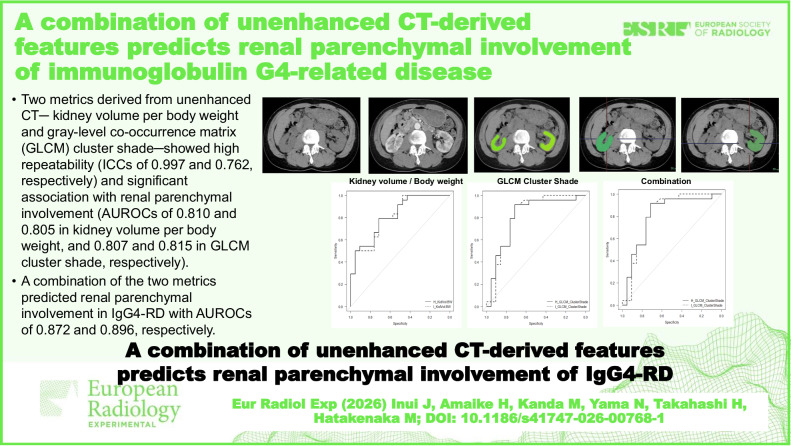

## Background

Immunoglobulin G4-related disease (IgG4-RD) is a systemic fibroinflammatory disorder involving many organs, such as the central nervous system, lacrimal glands, salivary glands, orbits, thyroid gland, thorax, breasts, pancreas and biliary tree, liver, retroperitoneum, aorta and vessels, prostate, skin, lymph nodes, and kidneys. Although the pathogenesis of the disease remains unclear, it is known that IgG4-associated inflammation and fibrosis are initiated by a combination of T-helper type 2 cells and regulatory T cells, leading to eosinophilia, elevation of serum IgG4 and immunoglobulin E, and fibrosis progression [1].

With respect to kidney involvement, the most common histologic manifestation of immunoglobulin G4-related kidney disease (IgG4-RKD) is tubulointerstitial nephritis, followed by glomerular disease, most often membranous glomerulonephritis [2]. The kidney involvement is divided into three locations: the renal parenchyma, renal pelvis and/or sinus, and perinephric region. Renal parenchymal lesions are the most common and show several imaging features, such as multiple nodular lesions, diffuse patchy infiltrative lesions, and single nodular lesions. Of these, the multiple nodular lesions are the most common pattern, and bilateral involvement is common [1]. On contrast-enhanced computed tomography (CE-CT), renal parenchymal lesions are mostly hypodense to the normal renal cortex in the early phase, *i.e*., the arterial or corticomedullary phase, and are progressively enhanced in the portal or delayed phase, and these lesions are usually not visible on unenhanced CT scans [3]

CT is usually the first-choice imaging modality for evaluating the spread of IgG4-RD, and CE-CT is also considered necessary to detect renal parenchymal involvement. However, patients with IgG4-RD are generally elderly, and their cases are often complicated with chronic kidney disease [4] and frequently associated with allergic diseases, such as bronchial asthma [5]. Accordingly, imaging methods allowing detection of renal parenchymal involvement without contrast media would be desirable. The utility of unenhanced magnetic resonance imaging (MRI) has also been reported recently, but this approach requires a long examination time to evaluate a wide range of sites, and its high examination cost prevents it from being a first-choice imaging modality.

In the present retrospective study, we used imaging analysis techniques such as segmentation and volume estimation, and texture analysis based on unenhanced CT, to identify imaging features that could predict renal parenchymal involvement in patients with IgG4-RD.

## Methods

Our institutional review board approved this retrospective study with an opt-out methodology (approval number of 372-92), and patient-informed consent was waived.

The datasets generated and/or analyzed during the current study are not publicly available due to the restrictions of the institutional review board, but are available from the corresponding author on reasonable request. When using data in other studies, new approval is needed.

### Participants

**Fig. 1:**
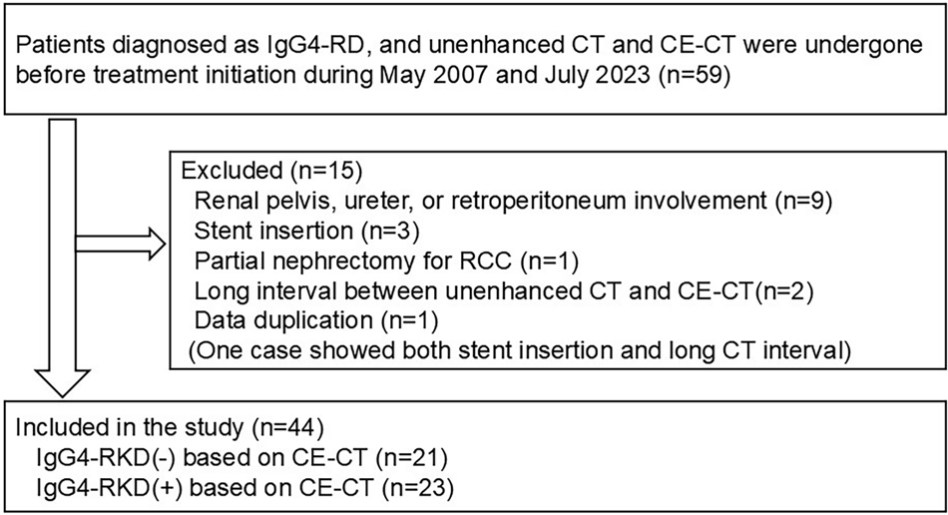
Summary of patient selection. CE-CT, Contrast-enhanced computed tomography; CT, Computed tomography; IgG4-RD, ImmunoglobulinG4-related disease; IgG4-RKD, Immunoglobulin G4-related kidney disease; RCC Renal cell carcinoma

Inclusion criteria were as follows: (1) IgG4-RD was diagnosed at our institute based on American College of Rheumatology/ European Alliance of Associations for Rheumatology classification criteria [6, 7]; and (2) both unenhanced CT and CE-CT covering the kidneys were conducted before treatment initiation between May 2007 and July 2023. The exclusion criteria were as follows: (1) renal pelvis, ureter, or retroperitoneum involvement; (2) stent insertion in the ureter; (3) past history of surgery for either kidney; (4) an interval of over one month between unenhanced CT and CE-CT. Fifty-nine patients were enrolled, and 15 were excluded, leaving 44 patients who were included in the analysis (Fig. 1). Some of the patients were also included in our previous report [8]; however, the two analyses had different purposes and methods.

### CT protocol

CT was performed using one of five scanners: Aquilion ONE, Aquilion PRIME, Aquilion Precision (Canon Medical Systems), LightSpeed VCT, or LightSpeed Ultra (GE Medical Systems). The tube voltage was 120 kVp, and the tube current was modulated automatically according to the patient’s body size. The slice thickness was usually 5 mm. In CE-CT, the patients were given 520 mg of iodine per kilogram of body weight with 300 or 370 mg/mL of nonionic contrast medium and a maximum dose of 135 mL per patient. The warmed contrast medium was administered intravenously with a power injector over 30–60 s through a 22-gauge catheter inserted into a cubital vein. CE-CT was obtained 90–180 s after the start of the contrast material injection, and arterial and/or delayed phase images were also obtained in some cases. CT was performed at other institutes in 6 cases; scanner type and detailed protocols could not always be obtained for those patients.

### Image analysis

#### Renal parenchymal involvement of IgG4-RD

For the 44 cases included in the study, renal parenchymal involvement was diagnosed based on the 2020 diagnostic criteria for IgG4-RKD [9] by two reviewers in consensus: a rheumatologist with over 15 years of experience (M.K.) and a diagnostic radiologist with over 30 years of experience (M.H.). As most cases did not undergo kidney biopsy and histological confirmation of kidney involvement was not obtained, the cases satisfying “abnormal renal radiologic findings” and “histologic findings in extra-renal organ(s)” were categorized as definite or probable for IgG4-RKD according to Saeki et al [9], and were defined as IgG4-RKD. As for “abnormal renal radiologic findings”, the cases showing multiple low-density lesions on CE-CT or a hypovascular solitary mass in the kidney, were evaluated as positive. The remaining two radiologic findings of “diffuse kidney enlargement” and “hypertrophic lesion of renal pelvic wall without irregularity of the renal pelvic surface”, were not included for evaluation, because renal pelvic lesions were already excluded in patient selection, and objective criteria of kidney enlargement were not defined by Saeki et al [9]. With respect to kidney enlargement, to propose an objective definition, we measured kidney volume-related metrics shown in the next paragraph.

#### Kidney volume measurement

**Fig. 2:**
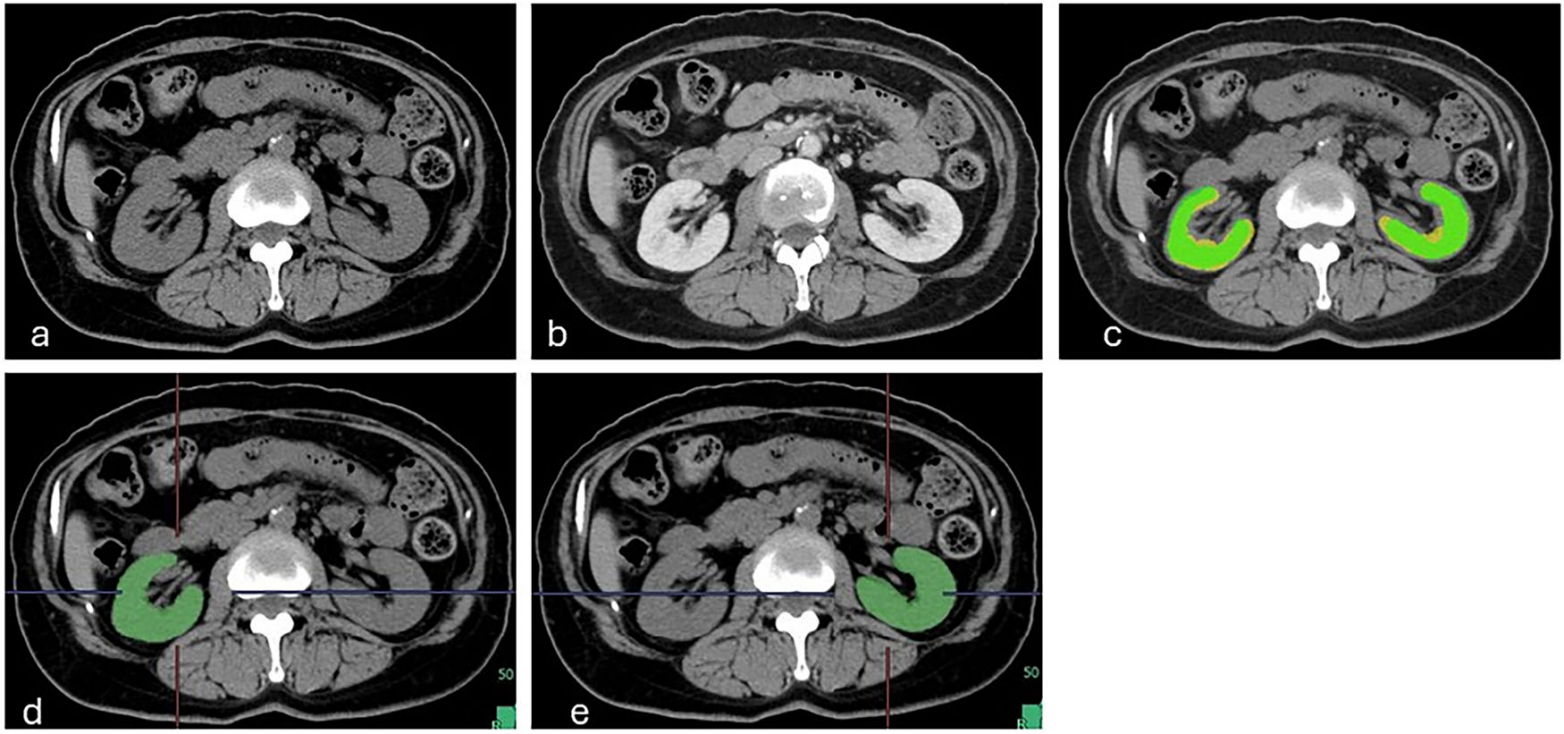
CT of an IgG4-RKD–negative case (female, 53 years). **a** Unenhanced CT of the kidney; **b** CE-CT of the kidney; **c** ROIs for texture extraction by MH (yellow) and JI (green); **d**, **e** segmentations (green) of the right and left kidneys by MH. CE-CT, Contrast-enhanced computed tomography; CT, Computed tomography; I gG4-RKD, Immunoglobulin G4-related kidney disease; ROI, Region of interest

**Fig. 3:**
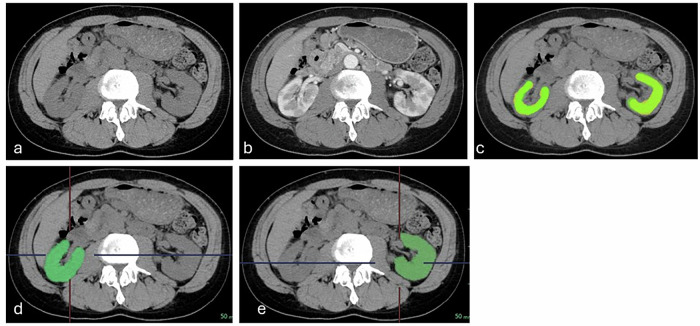
CT of an IgG4-RKD–positive case (male, 55 years). **a** Unenhanced CT of the kidney; **b** CE-CT of the kidney showing low-attenuation nodules in both kidneys; **c** ROIs for texture extraction by MH (yellow) and JI (green); **d**, **e** segmentations (green) of the right and left kidneys by MH. CE-CT, Contrast-enhanced computed tomography; CT, Computed tomography; I gG4-RKD, Immunoglobulin G4-related kidney disease; ROI, Region of interest

Two radiologists (J.I., a radiology resident with 4 years of experience, and M.H.) analyzed unenhanced CT images and measured kidney volume using SYNAPSE VINCENT® version 6.8 (FUJIFILM, Tokyo). The process is semiautomated, and the operator can make minor adjustments (Figs. 2 and 3) [10]. The mean value of both kidneys was assigned as kidney volume (mL). MH evaluated unenhanced CT at ≥ 1 month after the initial CE-CT reading to assess kidney involvement.

#### Texture extraction from unenhanced CT

Two radiologists (J.I. and M.H.) independently extracted texture metrics from unenhanced CT using the software package LIFEx version 7.70 [11]. The following settings were used: spatial resampling to 2 × 2 × 2 mm^3^, 64 gray levels, and intensity rescaling to the relative minimum and maximum. The regions of interest (ROIs) were assigned on three consecutive axial slices for both kidneys, and 3D image processing and segmentation were performed (Figs. 2 and 3). The mean values for both kidneys were used for each patient. The extracted and analyzed texture metrics are shown in Supplementary Table S1. MH extracted metrics from unenhanced CT at ≥ 2 months after the initial CE-CT reading to assess kidney involvement.

### Statistical analysis

Clinical data, including sex, age, body weight, and estimated glomerular filtration rate (eGFR), were obtained from hospital records. Group comparisons according to renal parenchymal involvement were performed using the χ^2^ test and *t*-test. Receiver operating characteristic (ROC) analysis was subsequently conducted for metrics that showed significant differences. The metrics showing areas under the ROC curve (AUROCs) ≥ 0.75 were chosen [12].

Three kidney-volume related parameters were analyzed: kidney volume (mL), kidney volume per body weight (mL/kg), and kidney volume per body surface area (mL/m^2^). For the kidney volume-related indexes and texture metrics, because many parameters were extracted, correlations between JI and MH were evaluated first, and those with an intraclass correlation coefficient (ICC) ≥ 0.7 were selected [13]. Then, the correlation with renal parenchymal involvement was evaluated using ROC analysis; the metrics with an AUROC ≥ 0.75 for both radiologists’ data were selected [12]; and binary logistic regression analysis was performed. In addition, ROCs of individual metrics with ICC ≥ 0.7 and an AUROC ≥ 0.75, as well as combined metrics derived from binary logistic regression, were compared between M.H. and J.I. To reduce the risk of overfitting, the least absolute shrinkage and selection operator (LASSO) 5-fold cross-validation was used.

Decision curve analysis was also performed to evaluate the clinical impact of our predictive markers. Net benefits were evaluated for the following: (1) a univariate model for kidney volume per body weight, (2) a univariate model for gray-level co-occurrence matrix (GLCM) cluster shade, and (3) a multivariate model (combination) incorporating both parameters. Each model was based on binomial logistic regression with IgG4-RKD as a binary outcome. The clinical usefulness was determined by comparing the net benefit of these models against the default strategies of “Treat All” and “Treat None” across the full spectrum of threshold probabilities. The analysis was performed by evaluators M.H., J.I., and MK using the “dcurves” package (v0.5.1) in R (v4.4.0).

Statistical analyses were performed with SPSS version 29 (International Business Machines Corporation), JMP student edition version 18 (SAS Institute Inc.), and R ver. 4.4.0 software. *p* values < 0.05 were considered statistically significant.

## Results

Of the 44 cases analyzed in this study, 23 were diagnosed as positive for IgG4-RKD, and 21 were diagnosed as negative (Fig. 1). In regard to the clinical data, age only exhibited a significant difference between the patients negative and those positive for renal parenchymal involvement. However, the AUROC was less than 0.75 (Table 1).

**Table 1:** Demographic and clinical characteristics of the patients

	IgG4-RKD		
	Negative (*n* = 21)	Positive (*n* = 23)	*p* value
Sex (male/female)	14 / 7	13 / 10	0.490
Age (years), mean ± SD	68.4 ± 9.6	57.9 ± 11.7	0.003 (AUROC < 0.75)
Weight (kg), mean ± SD	59.3 ± 10.7	58.3 ± 10.8	0.758
Estimated glomerular filtration rate (mL/min/1.73m^2^), mean ± SD	73.6 ± 21.7	71.8 ± 18.4	0.761

*IgG4-RKD* Immunoglobulin G4-related kidney disease, *SD* Standard deviation

**Fig. 4:**
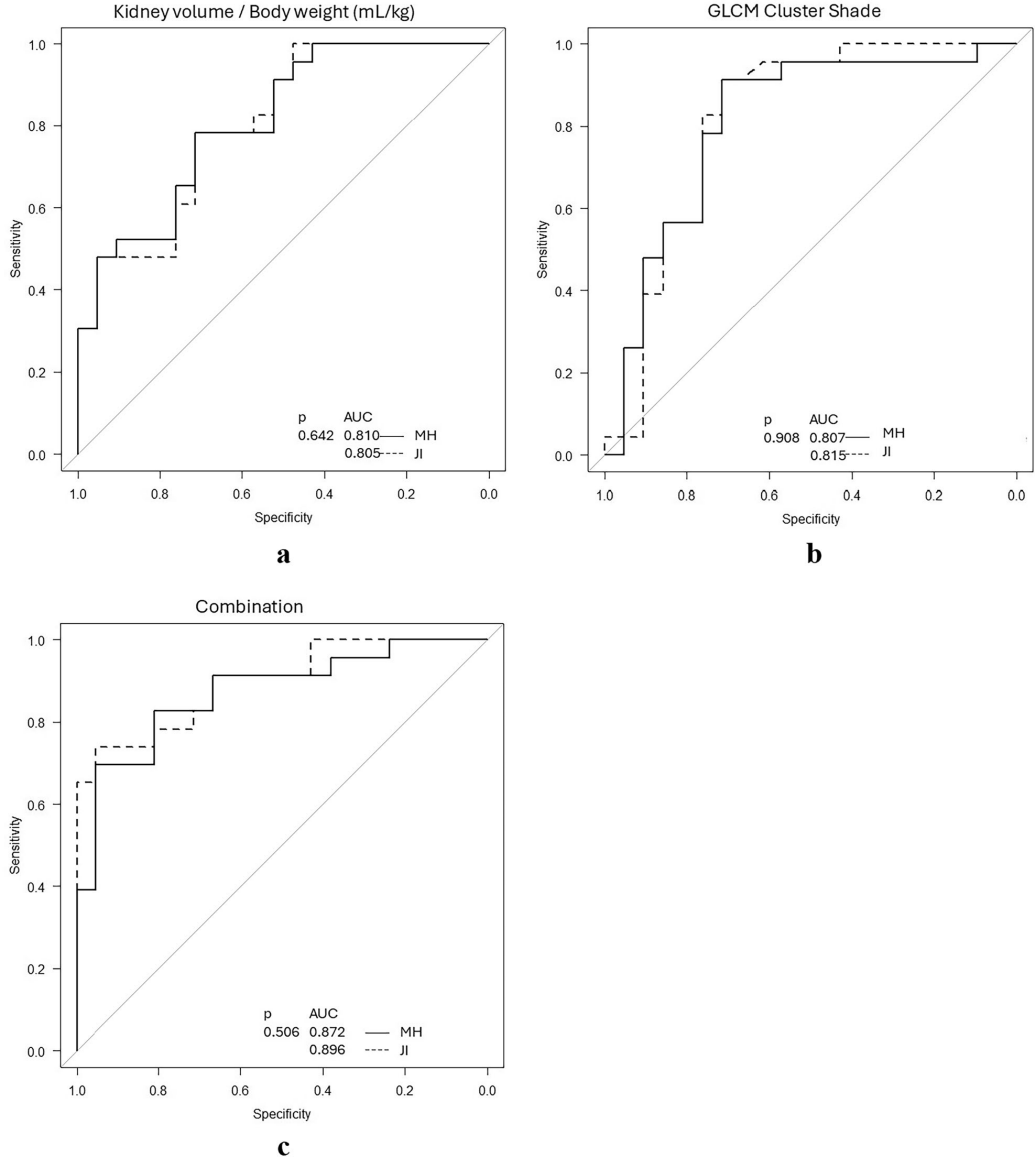
ROC generated by MH (solid line) and JI (dashed line) for kidney volume per body weight (**a**). GLCM cluster shade (**b**) and the combination of both metrics (**c**). No significant differences between MH and JI for any metrics were observed

The kidney volume, kidney volume per body weight, and kidney volume per body surface area showed high ICC values over 0.99, and kidney volume per body weight demonstrated the highest AUROCs for both radiologists: 0.810 and 0.805, respectively (Table 2 and Supplementary Table S2). Among texture metrics, 8 parameters demonstrated ICC ≥ 0.7. Of these, only GLCM cluster shade showed an AUROC ≥ 0.75 for both radiologists (Table 2 and Supplementary Table S2). Accordingly, kidney volume per body weight and GLCM cluster shade were analyzed for their ability to predict renal parenchymal involvement through binary logistic analysis with stepwise reduction. As a result, both kidney volume per body weight and GLCM cluster shade remained significant factors with AUROCs of 0.872 (sensitivity: 0.70, specificity: 0.95, positive predictive value: 0.94, negative predictive value: 0.74, accuracy: 0.82) and 0.896 (sensitivity: 0.74, specificity: 0.95, positive predictive value: 0.94, negative predictive value: 0.77, accuracy: 0.84), respectively. Finally, the ROCs of kidney volume per body weight, GLCM cluster shade, and the combination of both metrics were compared between M.H. and J.I., but no significant differences were observed. These results are summarized in Table 2 and Fig. 4. The cross-validation analyses showed virtually the same results for training and validation sets. Details were shown in Supplementary Results S3. Furthermore, to evaluate the model’s net benefit, decision curve analysis was also performed. All three models− kidney volume per body weight, GLCM cluster shade, and a combination of them−showed net benefit compared to treating all. However, using GLCM cluster shade only included the risk of no net benefit or demerit, especially at a range of high threshold probabilities ( ≥ 75%). Using kidney volume per body weight or a combination of kidney volume per body weight and GLCM cluster shade showed net benefit irrespective of threshold probability. Details were shown in Supplementary Results S4.

**Table 2:** Reproducibility and correlation of volume and texture metrics with Immunoglobulin G4-related kidney disease (IgG4-RKD)

Metrics	ICC	AUROC (95% CI)	
		MH (diagnostic radiologist)	JI (radiology resident)
Kidney volume	0.997	0.727 (0.579–0.875)	0.737 (0.591–0.883)
**Kidney volume/body weight**	**0.997**	**0.810 (0.684–0.935)**	**0.805 (0.678–0.933)**
Kidney volume/BSA	0.997	0.789 (0.657–0.921)	0.801 (0.671–0.931)
Vx	0.868	0.567 (0.395–0.740)	0.630 (0.463–0.798)
GLCM_correlation	0.793	0.694 (0.531–0.857)	0.637 (0.465–0.808)
**GLCM_cluster shade**	**0.762**	**0.807 (0.669–0.946)**	**0.815 (0.676–0.954)**
GLRLM run length non-uniformity	0.889	0.592 (0.421–0.764)	0.666 (0.503–0.828)
NGTDM_coarseness	0.856	0.536 (0.362–0.710)	0.579 (0.408–0.749)
NGTDM_busyness	0.817	0.607 (0.435–0.779)	0.639 (0.473–0.804)
GLSZM_gray level non-uniformity	0.771	0.563 (0.390–0.736)	0.514 (0.339–0.690)
GLSZM_zone size non-uniformity	0.761	0.747 (0.598–0.897)	0.831 (0.706–0.956)

*AUROC* Area under the receiver operating characteristic curve, *BSA* Body surface area, *CI* Confidence interval, *GLCM* Gray-level co-occurrence matrix, *GLRLM* Gray-level run length matrix, *GLSZM* Gray-level size zone matrix, *ICC* Intraclass correlation coefficient, *NGTDM* neighboring gray tone difference matrix, *Vx* PARAMS2_SPATIALFILTER_MeanFilter_Kernel size

Selected metrics are shown in bold

## Discussion

CT has been the first choice of imaging modality to assess the spread of IgG4-RD, and it is generally accepted that CE-CT is required to evaluate renal parenchymal involvement. To our knowledge, this study is the first to demonstrate that the combination of two unenhanced CT-related metrics—kidney volume per body weight and GLCM cluster shade—can predict renal parenchymal involvement of IG4-RD. In this analysis, the AUROCs for the two readers were 0.872 and 0.896.

Texture analysis has been increasingly applied in radiology because of its clinical utility; however, concerns remain regarding the reliability and reproducibility of many reported metrics. Therefore, we evaluated the reproducibility of each metric and adopted those with an ICC ≥ 0.7. As for ROI setting, assigning ROIs covering the entire kidney volume would be superior, but it is difficult to set ROIs at the edge of the kidneys, which may increase data variability. To increase reliability and also make the procedure easier, a method of assigning ROIs on three consecutive axial slices was chosen.

We did not include age as a metric because it did not satisfy the inclusion criteria. When Lasso analysis with 5-fold cross-validation was performed with three metrics−age, kidney volume per body weight, and GLCM Cluster Shade−age did not show significance, and overfitting was suspected, and no significant relation between age and kidney volume was observed (Supplementary Results S5). As the number of events in this cohort is 23 or 21, we consider that the number of explanatory variables is up to two, given the events per variable ratio ≥ 10.

GLCM cluster shade, which emphasizes locally shadowed areas and reflects the symmetry of the co-occurrence matrix, depends largely on the sum of row and column moments [14]. A higher absolute value indicates lower symmetry. In our analysis, the absolute GLCM cluster shade in IgG4-RKD–positive cases, was greater than that in negative cases (Supplementary Table S2). We interpret this finding as reflecting reduced textural symmetry in IgG4-RKD–positive kidneys, which likely include inflamed regions within the ROI.

Kidney enlargement is a well-recognized imaging feature of renal parenchymal involvement in IgG4-RKD, but no standardized method for its evaluation has been proposed [15]. The method proposed in this study is simple, semiautomatic, and highly reproducible [16]. Although combining kidney volume per body weight and GLCM cluster shade may appear complex for routine clinical use, LIFEx, while not approved for clinical application, has been increasingly used in research [17]. In practice, even measuring kidney volume per body weight could aid in predicting renal parenchymal involvement, based on the comparatively high AUROCs (> 0.8) obtained for this metric in our analysis. Setting threshold values for kidney volume per body weight (mL/kg) 2.54, sensitivity of 0.78, specificity of 0.71, positive predictive value of 0.75, negative predictive value of 0.75, and accuracy of 0.75 were performed (Supplementary Table S2). The software SYNAPSE VINCENT used in this study is approved for medical use in Japan and worldwide under the name SYNAPSE 3D, with regulatory clearances including European Union Medical Device Regulation—MDR and USA Food and Drug Administration–FDA 510(k).

We set the following exclusion criteria: (1) renal pelvis, ureter, or retroperitoneum involvement; (2) stent insertion in the ureter; (3) past history of surgery for either kidney; (4) interval of > 1 month between unenhanced CT and CE-CT. The reasons were as follows: (1) renal pelvis, ureter, or retroperitoneum involvement usually would be detectable on unenhanced CT. There may be a selection bias to exclude these patients, which may affect the evaluation of the patients with overlapping renal manifestations. For cases with mild imaging findings and/or overlapping findings, DWI may help reach a correct diagnosis; (2) a stent could affect kidney imaging characteristics; (3) surgical intervention may affect kidney volume-related indexes; (4) a long interval between images may change the status of renal parenchymal involvement. We assigned average values of both kidneys as the metrics for all patients because bilateral kidneys are usually involved [1, 18], and no patient showed unilateral kidney involvement in the present study.

Recent studies have reported that MRI, especially DWI, could detect renal parenchymal involvement without contrast media [18, 19]. However, MRI is not suitable for evaluating wide regions. Recently, diffusion-weighted whole-body MRI with background body signal suppression has been utilized clinically [20, 21]. Diffusion-weighted whole-body MRI with background body signal suppression has a large field-of-view and could detect kidney involvement, but a large field-of-view may deteriorate imaging quality, and, in some cases, it may be necessary to add conventional DWI for detailed kidney evaluation, prolonging the examination.

There are some limitations in this study. First, the number of patients analyzed was not sufficient to draw definitive conclusions. However, gathering a larger number of patients was not straightforward, as IgG4-RD is uncommon and only recently recognized as an entity. Second, this study was performed at a single institute. An external verification study with a large number of independent patients and with three or more readers would be desirable to yield a more clinically useful conclusion. Third, it would have been ideal to confirm renal parenchymal involvement histologically using biopsy specimens; however, this approach is invasive, and most of our cases did not undergo renal biopsy. Moreover, the purpose of this study was to predict renal parenchymal involvement using non-contrast imaging data. Therefore, the reference standard for renal parenchymal involvement was based on CE-CT findings, as determined by consensus between two board-certified readers. There may be a potential risk of using imaging-based criteria as both the reference standard and the comparator, and affecting diagnostic accuracy. However, in clinical practice, kidney biopsy for detecting disease involvement is too hard to perform for all patients. In general, imaging information based on CE-CT would be superior to that from unenhanced CT. The main objective of the study is to improve the detectability of renal parenchymal involvement by using non-contrast CT compared to using CE-CT. In this respect, we consider that this study could be useful in clinical practice, and using imaging reference could reduce study difficulty, and may facilitate a validation study in other institutes. Fourth, the present study included data from different scanners and protocols, which may affect metrics. To put it another way, the results of the present study, including several CT scanners and different type protocols, would be robust, though better results may be obtained when using the same scanners and protocols.

In conclusion, a combination of kidney volume per body weight and GLCM cluster shade derived from unenhanced CT can predict renal parenchymal involvement in IgG4-RD.

## Supplementary information

**Additional File 1:** **Table S1** The extracted and analyzed texture metric. **Table S2** Statistical characteristics of kidney volume per body weight and GLCM cluster shade by two observers.

## Data Availability

The datasets generated and/or analyzed during the current study are not publicly available due to the restrictions of the institutional review board, but are available from the corresponding author on reasonable request. When using data in other studies, new approval is needed.

## Abbreviations

AUROC: Area under the receiver operating characteristic curve
CE-CT: Contrast-enhanced computed tomography
CT: Computed tomography
GLCM: Gray-level co-occurrence matrix
ICC: Intraclass correlation coefficient
IgG4-RD: ImmunoglobulinG4-related disease
IgG4-RKD: Immunoglobulin G4-related kidney disease
MRI: Magnetic resonance imaging
ROC: Receiver operating characteristic
ROI: Region of interest
