# Supplementary material for: A combination of unenhanced CT-derived features predicts renal parenchymal involvement of immunoglobulin G4-related disease
Source: Eur Radiol Exp. 2026 Jun 25;10:101. doi: 10.1186/s41747-026-00768-1 (PMC13305061; doi:10.1186/s41747-026-00768-1)

# A combination of unenhanced CT-derived features predicts renal parenchymal involvement of immunoglobulin G4-related disease

## ELECTRONIC SUPPLEMENTARY MATERIAL

**Table S1** The extracted and analyzed texture metrics

PARAMS2_SPATIALFILTER_MeanFilter_KernelSize[vx]
GLCM_JointMaximum(IBSI:GYBY)
GLCM_JointAverage(IBSI:60VM)
GLCM_JointVariance(IBSI:UR99)
GLCM_JointEntropyLog2(IBSI:TU9B)
GLCM_JointEntropyLog10(IBSI:No)
GLCM_DifferenceAverage(IBSI:TF7R)
GLCM_DifferenceVariance(IBSI:D3YU)
GLCM_DifferenceEntropy(IBSI:NTRS)
GLCM_SumAverage(IBSI:ZGXS)
GLCM_SumVariance(IBSI:OEEB)
GLCM_SumEntropy(IBSI:P6QZ)
GLCM_AngularSecondMoment(IBSI:8ZQL)
GLCM_Contrast(IBSI:ACUI)
GLCM_Dissimilarity(IBSI:8S9J)
GLCM_InverseDifference(IBSI:IB1Z)
GLCM_NormalisedInverseDifference(IBSI:NDRX)
GLCM_InverseDifferenceMoment(IBSI:WF0Z)
GLCM_NormalisedInverseDifferenceMoment(IBSI:1QCO)
GLCM_InverseVariance(IBSI:E8JP)
GLCM_Correlation(IBSI:NI2N)
GLCM_Autocorrelation(IBSI:QWB0)
GLCM_ClusterTendency(IBSI:DG8W)
GLCM_ClusterShade(IBSI:7NFM)
GLCM_ClusterProminence(IBSI:AE86)
GLRLM_ShortRunsEmphasis(IBSI:22OV)
GLRLM_LongRunsEmphasis(IBSI:W4KF)
GLRLM_LowGreyLevelRunEmphasis(IBSI:V3SW)
GLRLM_HighGreyLevelRunEmphasis(IBSI:G3QZ)

GLRLM_ShortRunLowGreyLevelEmphasis(IBSI:HTZT)
GLRLM_ShortRunHighGreyLevelEmphasis(IBSI:GD3A)
GLRLM_LongRunLowGreyLevelEmphasis(IBSI:IVPO)
GLRLM_LongRunHighGreyLevelEmphasis(IBSI:3KUM)
GLRLM_GreyLevelNonUniformity(IBSI:R5YN)
GLRLM_RunLengthNonUniformity(IBSI:W92Y)
GLRLM_RunPercentage(IBSI:9ZK5)
NGTDM_Coarseness(IBSI:QCDE)
NGTDM_Contrast(IBSI:65HE)
NGTDM_Busyness(IBSI:NQ30)
NGTDM_Complexity(IBSI:HDEZ)
NGTDM_Strength(IBSI:1X9X)
GLSZM_SmallZoneEmphasis(IBSI:5QRC)
GLSZM_LargeZoneEmphasis(IBSI:48P8)
GLSZM_LowGrayLevelZoneEmphasis(IBSI:XMSY)
GLSZM_HighGrayLevelZoneEmphasis(IBSI:5GN9)
GLSZM_SmallZoneLowGreyLevelEmphasis(IBSI:5RAI)
GLSZM_SmallZoneHighGreyLevelEmphasis(IBSI:HW1V)
GLSZM_LargeZoneLowGreyLevelEmphasis(IBSI:YH51)
GLSZM_LargeZoneHighGreyLevelEmphasis(IBSI:J17V)
GLSZM_GreyLevelNonUniformity(IBSI:JNSA)
GLSZM_NormalisedGreyLevelNonUniformity(IBSI:Y1RO)
GLSZM_ZoneSizeNonUniformity(IBSI:4JP3)
GLSZM_NormalisedZoneSizeNonUniformity(IBSI:VB3A)
GLSZM_ZonePercentage(IBSI:P30P)
GLSZM_GreyLevelVariance(IBSI:BYLV)
GLSZM_ZoneSizeVariance(IBSI:3NSA)
GLSZM_ZoneSizeEntropy(IBSI:GU8N)

GLCM: Gray Level Co-occurrence Matrix
GLRLM: Gray Level Run Length Matrix
NGTDM: Neighbouring Gray Tone Difference Matrix

GLSZM: Gray Level Size Zone Matrix

GLCM_ClusterShade(S)=

$$\mu_x = \sum_{i=0}^{N_g-1} \sum_{j=0}^{N_g-1} i \cdot g(i,j)$$

$$\mu_y = \sum_{i=0}^{N_g-1} \sum_{j=0}^{N_g-1} j \cdot g(i,j)$$

$$S = \sum_{i=0}^{N_g-1} \sum_{j=0}^{N_g-1} (i + j - \mu_x - \mu_y)^3 \cdot g(i,j)$$

**Table S2** Statistical characteristics of kidney volume per body weight and GLCM cluster shade by two observers

| Metrics | Observer | IgG4-RKD | Mean ± SD | *p*-value | Threshold | Sensitivity | Specificity | PPV | NPV | Accuracy |
|---|---|---|---|---|---|---|---|---|---|---|
| Kidney volume / Body weight | MH | Positive | 2.89 ± 0.53 | < 0.001 | 2.54 | 0.78 | 0.71 | 0.75 | 0.75 | 0.75 |
| | | Negative | 2.31 ± 0.44 | | | | | | | |
| | JI | Positive | 2.88 ± 0.54 | < 0.001 | 2.54 | 0.78 | 0.71 | 0.75 | 0.75 | 0.75 |
| | | Negative | 2.29 ± 0.43 | | | | | | | |
| GLCM cluster shade | MH | Positive | -5,329 ± 2,250 | 0.002 | -2530 | 0.91 | 0.71 | 0.78 | 0.88 | 0.82 |
| | | Negative | -2,980 ± 2,384 | | | | | | | |
| | JI | Positive | -6,481 ± 2,410 | < 0.001 | -3702 | 0.91 | 0.71 | 0.78 | 0.88 | 0.82 |
| | | Negative | -3,569 ± 2,788 | | | | | | | |

There were significant differences between IgG4-RKD positive and negative in kidney volume per body weight and GLCM cluster shade for both readers. *GLCM* Gray-level co-occurrence matrix, *IgG4-RKD* Immunoglobulin G4-related kidney disease, *NPV* Negative predictive value, *PPV* Positive predictive value, *SD* Standard deviation.

**Supplementary Results S3**

**Cross-validation**

We analyzed data from MH and JI using Lasso with 5-fold cross-validation. As shown below, ROC curves from training and validation sets were virtually the same for both MH and JI, so, we interpreted our proposed method would be reliable.

MH

Training AUROC = 0.882

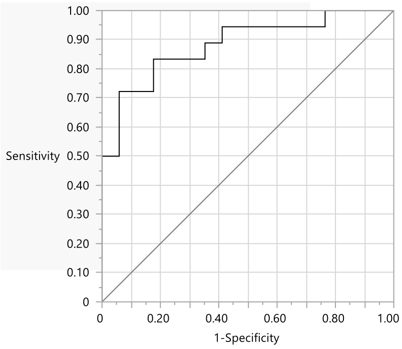


| Method | TP | FN | FP | TN | Sensitivity | Specificity | Precision | Accuracy |
|---|---|---|---|---|---|---|---|---|
| Fit Generalized | 15 | 3 | 4 | 13 | 0.8333 | 0.7647 | 0.7895 | 0.8 |

Validation AUROC = 0.80

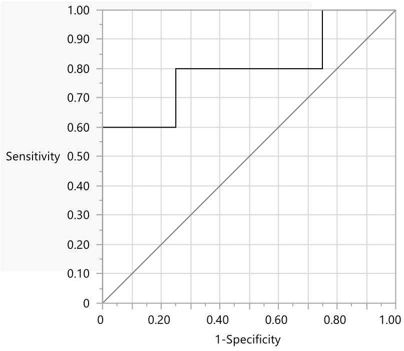


| Method | TP | FN | FP | TN | Sensitivity | Specificity | Precision | Accuracy |
|---|---|---|---|---|---|---|---|---|
| Fit Generalized | 4 | 1 | 1 | 3 | 0.8000 | 0.7500 | 0.8 | 0.7778 |

JI

Training AUROC=0.889

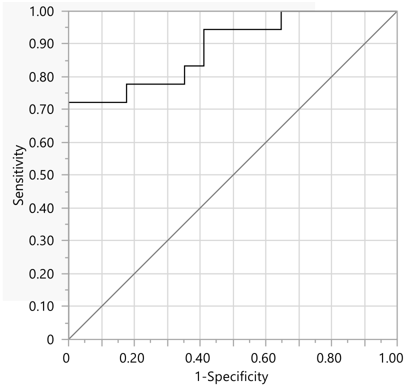


| Method | TP | FN | FP | TN | Sensitivity | Specificity | Precision | Accuracy |
|---|---|---|---|---|---|---|---|---|
| Fit Generalized | 14 | 4 | 3 | 14 | 0.7778 | 0.8235 | 0.8235 | 0.8 |

Validation AUROC=0.95

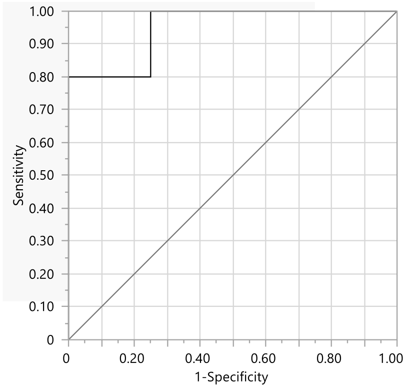


| Method | TP | FN | FP | TN | Sensitivity | Specificity | Precision | Accuracy |
|---|---|---|---|---|---|---|---|---|
| Fit Generalized | 4 | 1 | 1 | 3 | 0.8000 | 0.7500 | 0.8 | 0.7778 |

**Supplementary Results S4**

**Decision curve analysis for MH and JI data**

All three models− kidney volume per body weight, GLCM cluster shade, and combination of them−showed net benefit compared to treat all. However, using GLCM cluster shade only included risk of no net benefit or demerit especially at a range of high threshold probability (≥ 75%). Using kidney volume per body weight or combination of kidney volume per body weight and GLCM cluster shade showed net benefit irrespective of threshold probability.

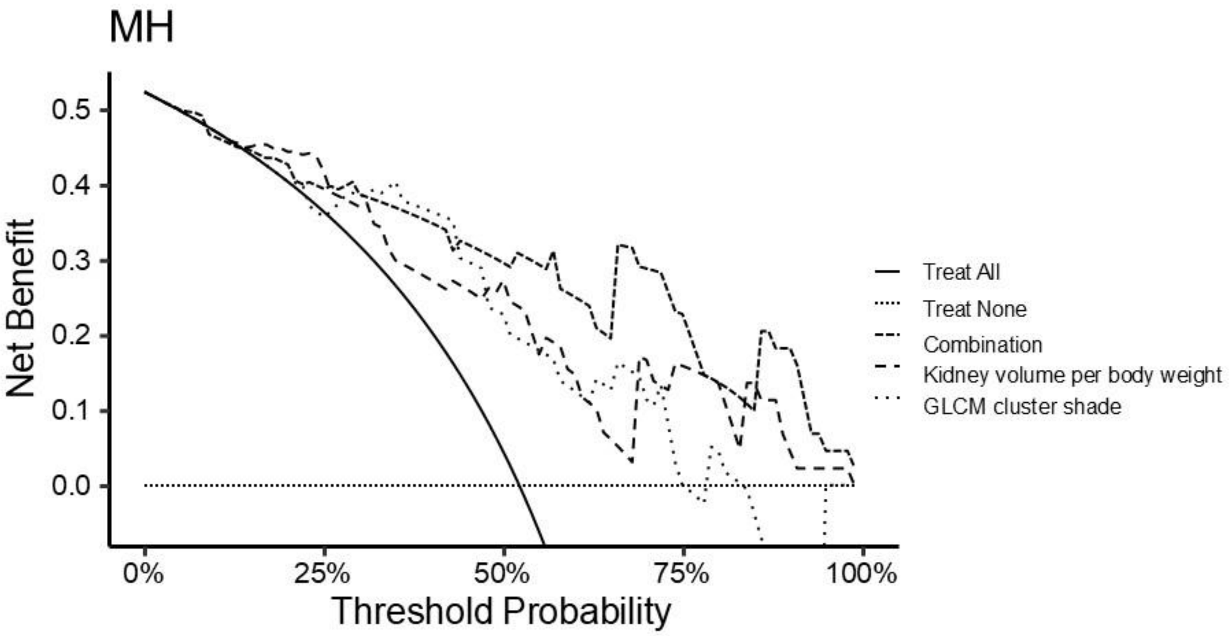


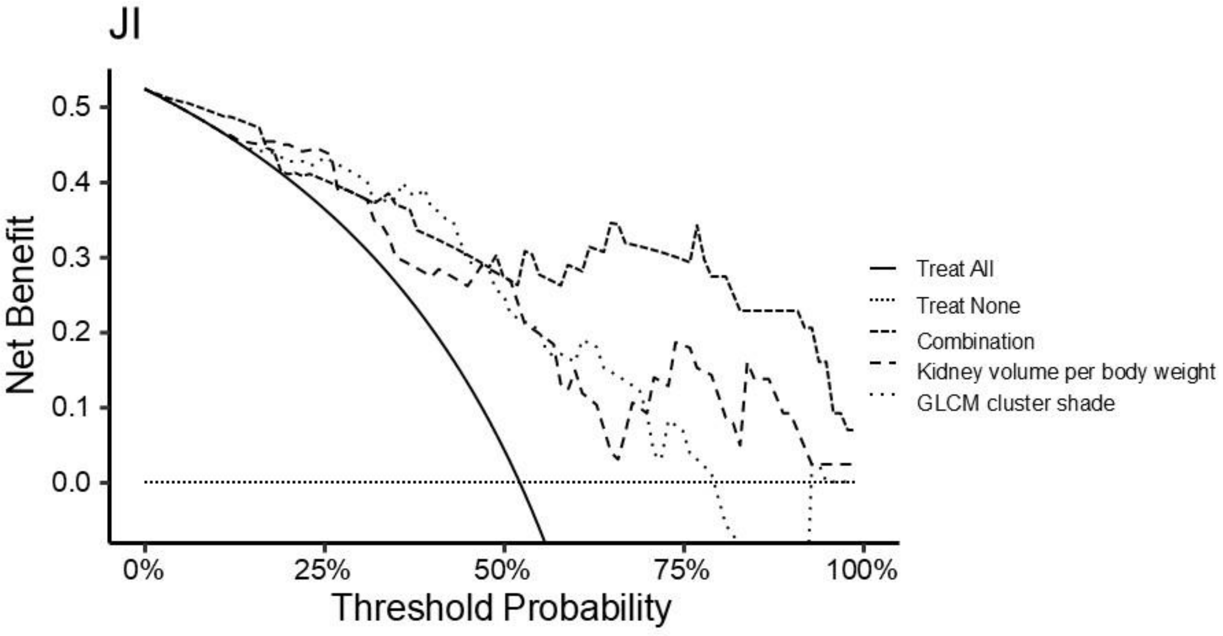

**Supplementary Results S5**

When Lasso analysis with 5-fold cross-validation was performed with three metrics—age, kidney volume per body weight, and GLCM Cluster Shade. The age did not show significance and overfitting was suspected.

IJ

| Source | Nparm | DF | Wald $\chi^2$ | Prob > $\chi^2$ |
|---|---|---|---|---|
| Kidney volume per body weight | 1 | 1 | 10.096671 | 0.0015* |
| GLCM cluster shade | 1 | 1 | 7.3442024 | 0.0067* |
| Age | 1 | 1 | 3.640681 | 0.0564 |

Training AUROC=0.944

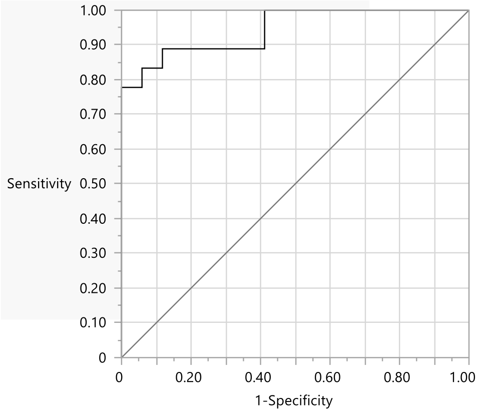


Validation AUROC=0.800

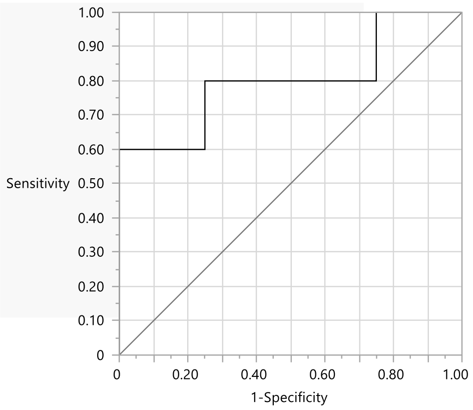


Training

| Method | TP | FN | FP | TN | Sensitivity | Specificity | Precision | Accuracy |
|---|---|---|---|---|---|---|---|---|
| Fit Generalized | 16 | 2 | 2 | 15 | 0.8889 | 0.8824 | 0.8889 | 0.8857 |

Validation

| Method | TP | FN | FP | TN | Sensitivity | Specificity | Precision | Accuracy |
|---|---|---|---|---|---|---|---|---|
| Fit Generalized | 4 | 1 | 1 | 3 | 0.8000 | 0.7500 | 0.8 | 0.7778 |

Age *versus* kidney volume. No significant relation was observed between age and kidney volume.

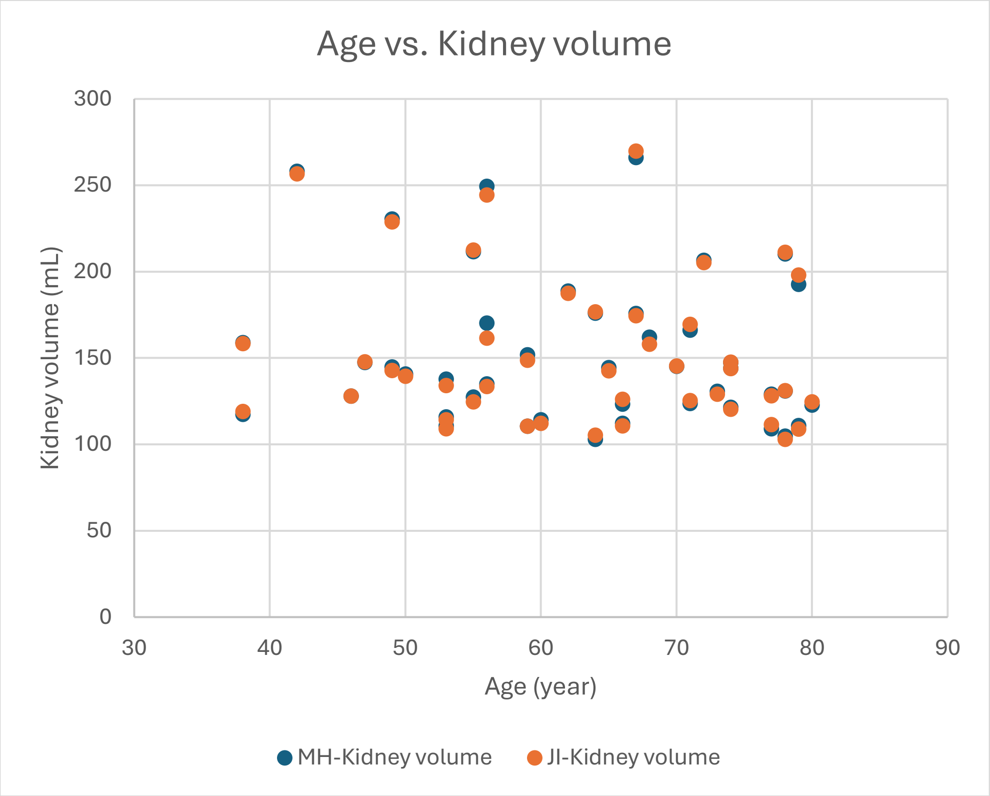

Supplement: Supplementary file 1 — Additional File 1: Table S1 The extracted and analyzed texture metric. Table S2 Statistical characteristics of kidney volume per body weight and GLCM cluster shade by two observers. [file 41747_2026_768_MOESM1_ESM.pdf]
